# The comparative genomic analysis provides insight into the divergent inhibitory activity metabolites in pathogen-driven three *Pseudomonas palleroniana* strains against primary pathogens of *Pseudostellaria heterophylla*

**DOI:** 10.1186/s12864-025-11527-8

**Published:** 2025-04-02

**Authors:** Chunfeng Huang, Xiaoai Wang, Yanping Gao, Xue Jiang, Lingling Wang, Xiaohong Ou, Yanhong Wang, Tao Zhou, Qing-Song Yuan

**Affiliations:** 1 Guizhou Key Laboratory for Germplasm Innovation and Resource-Efficient Utilization of Dao-di Herbs, Resource Institute for Chinese & Ethnic Materia Medica, Guizhou University of Traditional Chinese Medicine, Guiyang, 550025 China; 2 School of Food and Biological Engineering, Jiangsu University, Zhenjiang, 212013 China; 3 State Key Laboratory for Quality Ensurance and Sustainable Use of Dao-di Herbs, National Resource Center for Chinese Materia Medica, China Academy of Chinese Medical Sciences, Beijing, 100700 China

**Keywords:** *Pseudomonas*, Biocontrol, *Pseudostellaria heterophylla*, Whole genome sequencing, Multiple pathogens

## Abstract

*Pseudostellaria heterophylla* (Miq.) Pax ex Pax et Hoffm. is a member of the *Caryophyllaceae* family, in which dried tuberous root is the well-known traditional Chinese medicine (TCM) and a widespread food ingredient in Asia. In recent years, the large-scale cultivation of *P. heterophylla* has led to frequent infectious diseases caused by multiple pathogens. However, efficient and safe approaches for preventing and managing P. heterophylla diseases have become urgent for this high-quality industrial development. Herein, a culturable microbiome of diseased *P. heterophylla* rhizosphere soil was constructed, and the broad-spectrum antifungal activity of *Pseudomonas* was screened. Three *P. palleroniana* strains, B-BH16-1, B-JK4-1, and HP-YBB-1B, were isolated and identified with vigorous antifungal activity by confrontation method. We employed the PacBio RS II single-molecule real-time (SMRT) sequencing and Illumina sequencing methods to obtain the genome of these three isolates. Phylogenetic, synteny, and ANI analysis showed that the lineage between strain B-JK4-1 with B-BH16-1 or HY-YBB-1B was closer than that between strain B-BH16-1 with HP-YBB-1B. The comparative genome of strains B-BH16-1, B-JK4-1, and HP-YBB-1B showed marked differences in secondary metabolite biosynthesis genes among these three P. palleroniana strains. Strain B-BH16-1, B-JK4-1, and HP-YBB-1 produced tolaasin I/tolaasin F (23 genes), sessilin A (37 genes), and putisolvin (39 genes), respectively. CAZyme analysis showed that 126, 129, and 127 CAZymes were identified in strains B-BH16-1, B-JK4-1, and HP-YBB-1B genomes, which genes in auxiliary activities (AA), carbohydrate esterases (CE), and glycosyl transferases (GT) categories were different among these three strains. These results provide new insights into the divergent antifungal metabolites in pathogen-driven three *P. palleroniana* strains against primary pathogens of *Pseudostellaria heterophylla*.

**Supplementary Information:**

The online version contains supplementary material available at 10.1186/s12864-025-11527-8.

## Introduction

*Pseudostellaria heterophylla* (Miq.) Pax ex Pax et Hoffm. is a member of the *Caryophyllaceae* family, which dried tuberous root is a well-known traditional Chinese medicine (TCM) and a widespread food ingredient in Asia for more than 100 years due to its anti-fatigue and immune-enhancing properties [1–3]. *P. heterophylla* is predominantly cultivated in Guizhou, Fujian, Anhui, and Jiangsu provinces in China, for which an annual demand is estimated between 7,000 and 8,000 tons [4]. In recent years, the large-scale cultivation of *P. heterophylla* has led to frequent infectious diseases caused by multiple pathogens associated with twenty kinds of pathogen species, such as *Fusarium* spp. and *Alternaria alternata* [5, 6]. Our previous research found that *F. oxysporum*, *A. alternata*, *Arcopilus aureus*, *Botrytis cinerea*, *Nemania diffusa*, *Whalleya microplaca*, *and Cladosporium cladosporioides* were identified as the dominant pathogens of foliar disease in *P. heterophylla* [7]. Among these pathogens, *F. oxysporum* and *A. alternata* are particularly common, and their severity results in several economic losses in crops and fruit, including bananas, tomatoes, melons, and watermelons [8–10]. Many previous studies have shown that pathogens produce massive toxins to promote colonization and invade the host, severely reducing food quality and TCM [11, 12]. Therefore, the efficient and safe prevention and management of *P. heterophylla* diseases has become urgent for this industrial high-quality development.

In this decade, to solve the increasingly pronounced conflict between disease prevention and ecological protection, the development and utilization of broad-spectrum antifungal strains represent an effective strategy to minimize the reliance on chemical agents and mitigate environmental pollution. The rhizosphere microorganisms, particularly plant growth-promoting rhizobacteria (PGPR), are widely applied in agricultural systems due to their complexity and variability habitats exhibiting strong environmental adaptability and diverse biological control mechanisms [13]. Previous research indicates that rhizosphere microorganisms can effectively colonize plant roots and enhance plant resistance through a range of mechanisms, including phosphate solubilization, plant growth regulators production, nitrogen fixation, ethylene metabolism, and antifungal metabolites and iron carriers production [14, 15]. Consequently, rhizosphere microorganisms are increasingly recognized as an environmentally sustainable alternative to chemical pesticides in agricultural practices.

*Pseudomonas*, one kind of plant growth-promoting rhizobacteria composing more than 100 species, has attracted considerable attention in the biological control of plant diseases [16, 17]. They exhibit a diversity of antifungal compounds and possess the capability to synthesize a wide array of secondary metabolites, including hydrogen cyanide (HCN), phenazine, phenazine-1-carboxylic acid, 2-acetaminophenol, aeruginaldehyde, pyrrolnitrin, 2,4-diacetyl phloroglucinol, pyocyanin, and lipopeptides [18–21]. For instance, the *P. aeruginosa* strain Gxun-2 can significantly inhibit the incidence of Fusarium wilt in bananas caused by *F. oxysporum* f.sp. *cubense* Tropical Race 4 (FOC TR4) [22]. Evenly, *P. fluorescen*s synthesizes antifungal antibiotics that directly inhibit pathogen growth and specifically target the pathogen’s infection factors [23]. Our previous research found that *P. palleroniana* B-BH16-1 directly antagonized multiple pathogens and indirectly disrupted the pathogen virulence factor biosynthesis to enhance disease suppression and improve yields of *Pseudostellaria heterophylla* [1]. In addition, *Pseudomonas* can inhibit the colonization or proliferation of pathogens by inducing systemic resistance in the host. For instance, *P. putida* strain RRF3 enhances disease resistance in rice by modulating the rhizosphere’s root transcriptome and chemical composition to activate the plant’s defense response [24]. Numerous Pseudomonas, as plant growth-promoting rhizobacteria, can also secrete plant growth regulators that enhance the plants’ capacity to resist pathogens [25]. The *P. aeruginosa* exhibits a pronounced plant growth-promoting effect and significant resistance activity against *A. alternata*, *Aspergillus flavus*, and *F. oxysporum* [16]. The *Pseudomonas fluorescens* strain SS101 produces volatile organic compounds that facilitate the growth of *Arabidopsis thaliana*, promote chlorophyll accumulation and lateral root development, and enhance disease resistance [26]. Consequently, *Pseudomonas* demonstrates considerable potential for agricultural biological fungicide or fertilizer application.

Our previous research demonstrated that the incidence process of Fusarium wilt induced the enrichment of *Pseudomonas* in the rhizosphere of *P. heterophylla* [27], which significantly enhanced the disease resistance via the release of volatile organic compounds [1, 28]. A culturable microbiome of *P. heterophylla* rhizosphere soil was constructed, and the broad-spectrum antifungal activity of *Pseudomonas* was screened. The confrontation method identified three isolates (B-BH16-1, B-JK4-1, and HP-YBB-1B) of *P. palleroniana* with strong antifungal activity. We employed the PacBio RS II single-molecule real-time (SMRT) sequencing and Illumina sequencing methods to obtain the genome of these three strains. In addition, we predicted the carbohydrate-active enzymes (CAZymes) and candidate antifungal metabolites based on genome sequence, which provides new insights into the divergent antifungal metabolites in pathogen-driven three *P. palleroniana* strains against primary pathogens of *Pseudostellaria heterophylla*.

## Materials and methods

### Construction of culturable Microbiome of *P. heterophylla* rhizosphere soil

Rhizosphere soil of diseased *P. heterophylla* was collected from cultivation base in Huangping County (N27°4’21”, E108°8’0”) and Majiang County (N26°29’28”, E107°35’22”) in Guizhou, China. Isolation of culturable bacteria from rhizosphere soil of diseased *P. heterophylla* was conducted using a dilution plate method on Luria-Bertani (LB) agar medium (NaCl 5 g/L, tryptone 10 g/L, and yeast extract 5 g/L) according to our previous research [27]. Briefly, five g collected rhizosphere soil samples were sonicated in 20 ml of PBS solution for 30 min, and an aliquot (1 ml) of the suspension was diluted ten times. An aliquot (100 µl) of the dilution suspension was coated onto LB medium and cultured at 25℃ for 2 days, and then, individual colonies were isolated and stored at -80℃ in 20% glycerol. The culturable microbiome described below identified the B-BH16-1, B-JK4-1, and HP-YBB-1B isolates with antifungal activity.

### Determination of antifungal activity in vitro

Eleven dominant pathogens including Epicoccum nigrum, Chaetomium globosum, Fusarium oxysorum, F. tricinctum, Alternaria alternata, Chizophyllum commune, Rhizopus oryzae, Botryotinia fuckeliana, Lasiodiplodia theobromae, Coprinellus xanthothrix, and Whalleya microplaca identified in our previous study, are selected as targets pathogens for antifungal activity analysis. A confrontment assay was conducted according to our previous study [28]. Briefly, the fresh mycelium cake of pathogen was inoculated on the center of potato dextrose agar (PDA) medium. Then 10 µL of fresh cells (OD_600_ = 1) of B-BH16-1, B-JK4-1, HP-YBB-1B, and Escherichia coli DH5α (as a control) were inoculated at a distance of 2 cm from the center according with a clockwise. All plates were cultured at 25℃ in darkness. After five days of incubation, the mycelial semidiameter of a pathogen in each treatment was measured. The inhibition rate was calculated as follows: Inhibition rate (%) = (the mycelia semidiameter of DH5α − the mycelia semidiameter of treatment)/the semidiameter of DH5α × 100. All experimental strains were performed in triplicate.

### Identification of strains B-BH16-1, B-JK4-1, and HP-YBB-1B

The strain was initially activated in an LB medium to cultivate a single colony, which was subsequently inoculated into a fresh LB medium using a sterile toothpick. It was incubated at 37°C for 24 hours with shaking at 180 rpm and then centrifuged at 11,000 rpm for five minutes to collect the bacterial cells. Genomic DNA extraction was performed according to the instructions provided by the Wizard^®^ Genomic DNA Purification Kit (Promega). The V3V4 rRNA fragment was amplified by universal paired primers (341F: 5’-ACTCCTACGGGAGGCAGCAG-3’ and 806R: 5’-GGACTACHVGGGTWTCTAAT-3’). The PCR products were purified and sequenced by Shanghai Shenggong Bioengineering Co. Ltd. The resulting sequences were aligned with the NR database available at https://www.ncbi.nlm.nih.gov/. Finally, similar sequences were selected to construct the phylogenetic tree by the adjacency method using Mega (ver. 11.0), which was bootstrapped 1,000 times.

### Genome sequencing and assembly

CTAB methods extracted the genomic DNA, and the more than 15 kb high-quality DNA was used for library preparation. The combination of PacBio RS II single-molecule real-time sequencing (SMRT) and the Illumina sequencing methods (Majorbio Bio-Pharm Technology Co., Ltd., Shanghai, China) was employed for genome sequencing of strains B-BH16-1, B-JK4-1, HP-YBB-1B. The NEXTFLEX Rapid DNA-Seq Kit was utilized to prepare the library, which was subsequently sequenced in a paired-end manner (2 × 150 bp) on an Illumina NovaSeq 6000 platform. The raw data generated from the Illumina sequencing machine were trimmed using fastp (ver. 0.23.0) to eliminate reads with low sequencing quality, high N content, and short-length reads. The HiFi reads were generated from the PacBio Sequel IIe platform. Genome assembly was performed by Unicycler (ver. 0.4.8) based on the HiFi reads [29], and then the Illumina sequence corrected the genome reads using Pilon (ver. 1.22).

### Genome annotation and bioinformatics analysis

The gene prediction in the genome was performed by Glimmer (http://ccb.jhu.edu/software/glimmer/index.shtml) [30], GeneMarkS [31], and Prodigal [32]. The tRNA was predicted by tRNAscan-SE (v2.0) [33], and rRNA was determined using barrnap (ver. 0.9, https://github.com/tseemann/barrnap) [34]. The gene function was annotated by homolog-based method sequence alignment tools using BLASTP, Diamond, and HMMER against NR, Swiss-Prot, Pfam, GO, COG, KEGG, and CAZY databases. Additionally, the biosynthetic gene cluster responsible for secondary metabolites was predicted by antiSMASH (ver. 5.1.2) against MiBIG database.

The VEEN diagrams were constructed to visualize shared and unique genes among three strains. The synteny and ANI analyses were performed on the Majorbio Cloud Platform (cloud.majorbio.com) using default parameters. The species designation of strains B-BH16-1, B-JK4-1, and HP-YBB-1B was conducted on Ribosomal Multilocus Sequence Typing (rMLST, https://pubmlst.org/bigsdb?db=pubmlst_rmlst_seqdef_kiosk) analysis and Type Strain Genome Server (TYGS, https://tygs.dsmz.de/user_requests/new) based on genome sequence at default parameters [35]. The histogram and heatmap were visualized by Origin software (Version 2018, Origin Lab Inc., Northampton, MA, USA) to illustrate the number of genes of CAZmyes.

## Results

### Broad and diversity spectrum antagonistic activity of strains B-BH16-1, B-JK4-1, and HP-YBB-1B against dominant pathogens of *P. heterophylla*

**Fig. 1:**
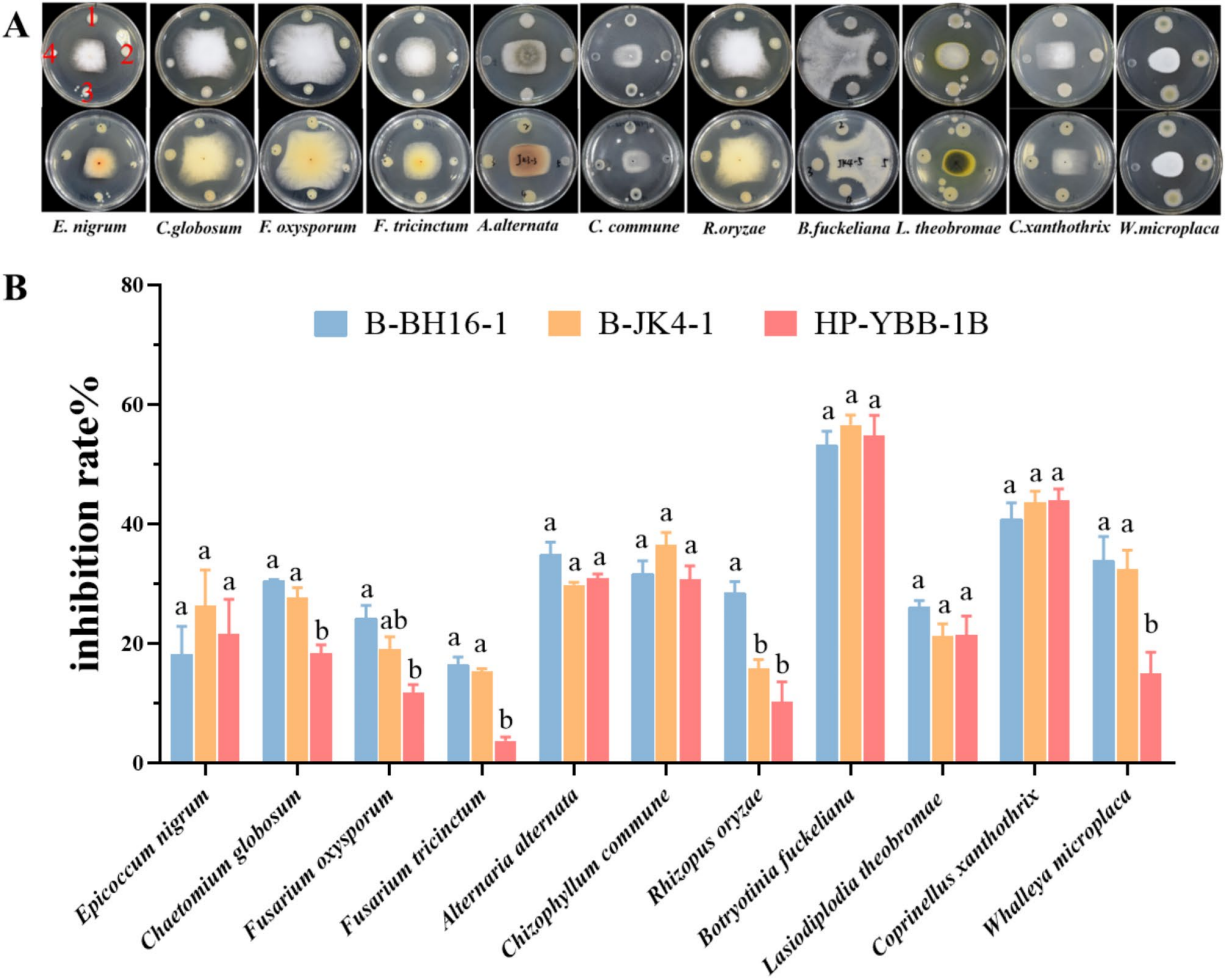
The antifungal activity of *P. palleroniana* strains B-BH16-1, B-JK4-1, and HP-YBB-1B against eleven pathogens in *P. heteraphylla*. 1, 2, 3, and 4 were represent B-BH16-1, B-JK4-1, HP-YBB-1B, and DH5α. Different letters represent the significant differences at the *P* = 0.05 level

Seven strains were isolated from the rhizosphere soil of diseased *P. heterophylla*. The confrontment assay challenged the eleven pathogens of *P. heterophylla*, and the inhibition rates were also measured. After five days of incubation, the strains B-BH16-1, B-JK4-1, and HP-YBB-1B showed significant antagonistic activity against all eleven pathogens (Fig. 1A and B). Among them, strains B-BH16-1, B-JK4-1, and HP-YBB-1B displayed the highest antagonistic activity on *B. fuckeliana* with inhibition rates of 53.24%, 56.55%, and 54.75%, respectively, in contrast with other pathogens. They showed the lowest antagonistic activity on *F. tricinctum* with inhibitory rates of 13.07%, 12.00%, and 3.71%, respectively. Notably, the inhibitory ability of strain HP-YBB-1B against pathogens is lower than that of strains B-BH16-1 and B-JK4-1, particularly against *C. globosum*, *F. oxysporum*, *F. tricinctum*, and *W. microplaca*.

### Genome sequencing and assembly of strains B-BH16-1, B-JK4-1, and HP-YBB-1B

**Fig. 2:**
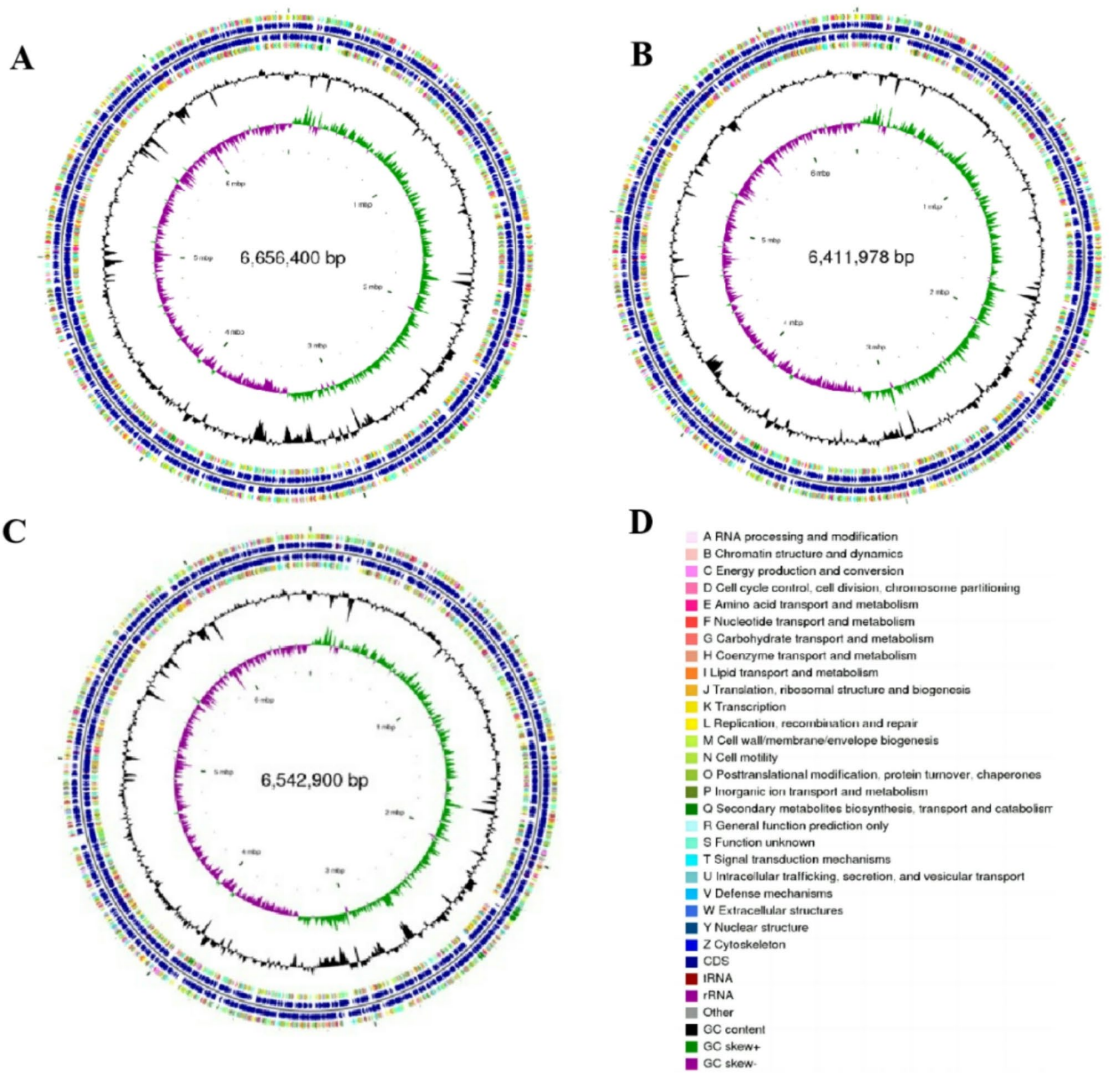
The genome maps of *P. palleroniana* strains B-BH16-1 (**A**), B-JK4-1 (**B**), and HP-YBB-1B (**C**). From outer to inner circles, the first and fourth circles represent coding sequences (CDS) on positive and negative strands, respectively, with distinct colors indicating different COG functional classifications. The second and third circles illustrate tRNA and rRNA on both positive and negative strands. The fifth circle denotes GC content; regions in this section indicate higher GC content than the average GC content of the entire genome, where a higher peak value signifies a more significant deviation from this average; conversely, regions with lower GC content than that average are represented in another section. The sixth circle displays GC skew values

To comprehend the phylogeny and function of strains B-BH16-1, B-JK4-1, and HP-YBB-1B, their complete genomes were sequenced and assembled by integrating PacBio RS II single-molecule real-time sequencing and the Illumina sequencing methods. Totally, 127,013, 172,200, and 116,113 reads were generated in strains B-BH16-1, B-JK4-1, and HP-YBB-1B, respectively, which were assembled to 6.66 Mb, 6.51 Mb, and 6.54 Mb circular genome (Table 1; Fig. 2). GC content is 60.29%, 60.45%, and 60.40%, respectively. 5,989, 5,746, and 5,877 protein-coding genes were predicted through the genome of strains B-BH16-1, B-JK4-1, and HP-YBB-1B, respectively. Total noncoding genes were 165 (19 rRNA, 70 tRNA, and 75 sRNA), 166 (19 rRNA, 70 tRNA, and 76 sRNA), and 175 (19 rRNA, 79 tRNA, and 76 sRNA) in strains B-BH16-1, B-JK4-1, and HP-YBB-1B, respectively. The tandem repeat sequences were 29,674 bp, 21,861 bp, and 26,998 bp, accounting for 0.50%, 0.38%, and 0.47% of the genome size of strains B-BH16-1, B-JK4-1, and HP-YBB-1B, respectively (Table 2).

**Table 1:** Genomic characteristics of *P. palleroniana* strains B-BH16-1, B-JK4-1, and HP-YBB-1B

Item	B-BH16-1	B-JK4-1	HP-YBB-1B
Genome size	6,656,400	6,511,978	6,542,900
Number of total reads	127,013	172,200	116,113
GC content (%)	60.29	60.45	60.40
Number of CDSs	5989	5746	5877
Number of rRNAs	19	19	19
Number of tRNAs	70	70	79
Number of sRNAs	75	76	76
Number of Chromo	1	1	1
Gene cluster	17	16	16
Genes assigned to NR	5854	5623	5755
Genes assigned to GO	4319	4274	4272
Genes assigned to KEGG	3239	3191	3170
Genes assigned to COG	5130	4996	5065
Genes assigned to Pfam	5079	4943	5029

**Table 2:** Statistical analysis of tandem repeat sequences (TRs)

Sample Name	Repeat No.	Total Len (bp)	In genome (%)
B-BH16-1	56	29,674	0.50
B-JK4-1	45	21,861	0.38
HP-YBB-1B	46	26,998	0.47

### Genome annotation

**Fig. 3:**
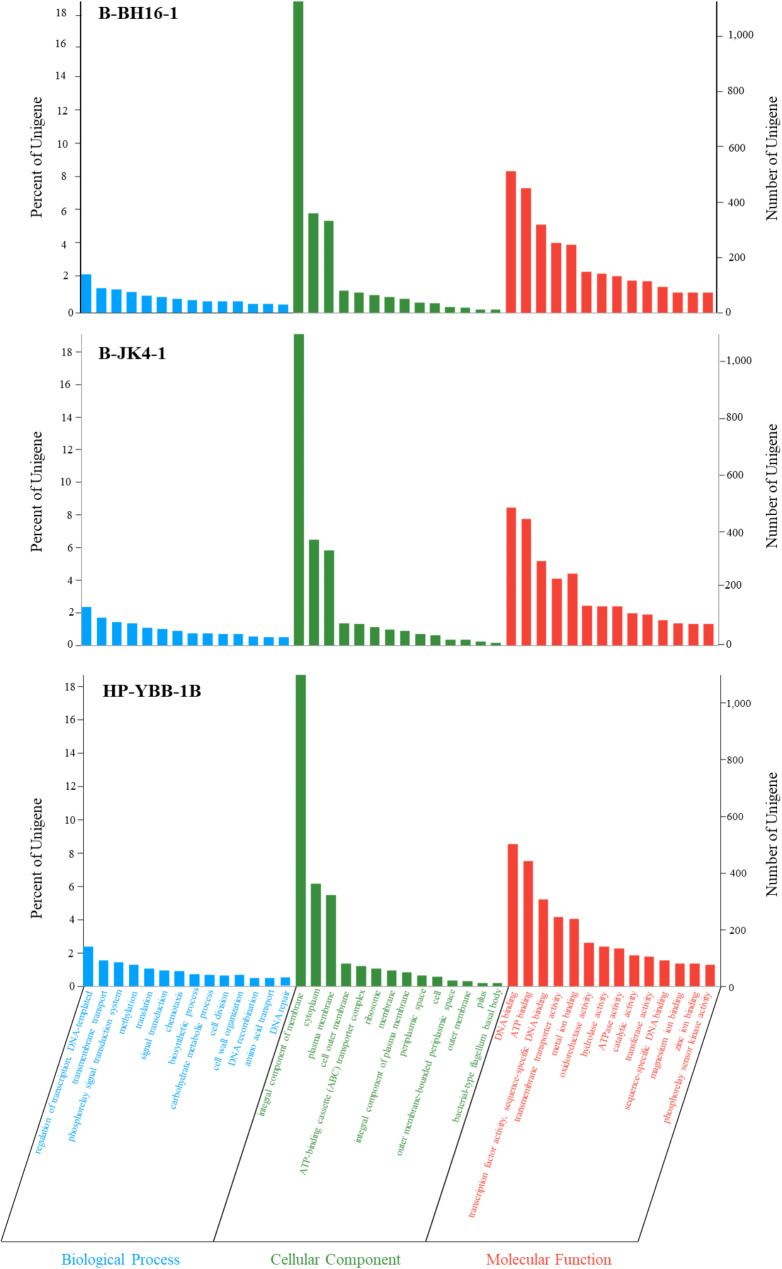
The functional annotation of the genome Gene Ontology (GO) for *P. palleroniana* strain B-BH16-1, B-JK4-1, and HP-YBB-1B. The x-axis denotes three branches of GO: Biological Process (BP), Cellular Component (CC), and Molecular Function (MF), along with their further classification at level 2

**Fig. 4:**
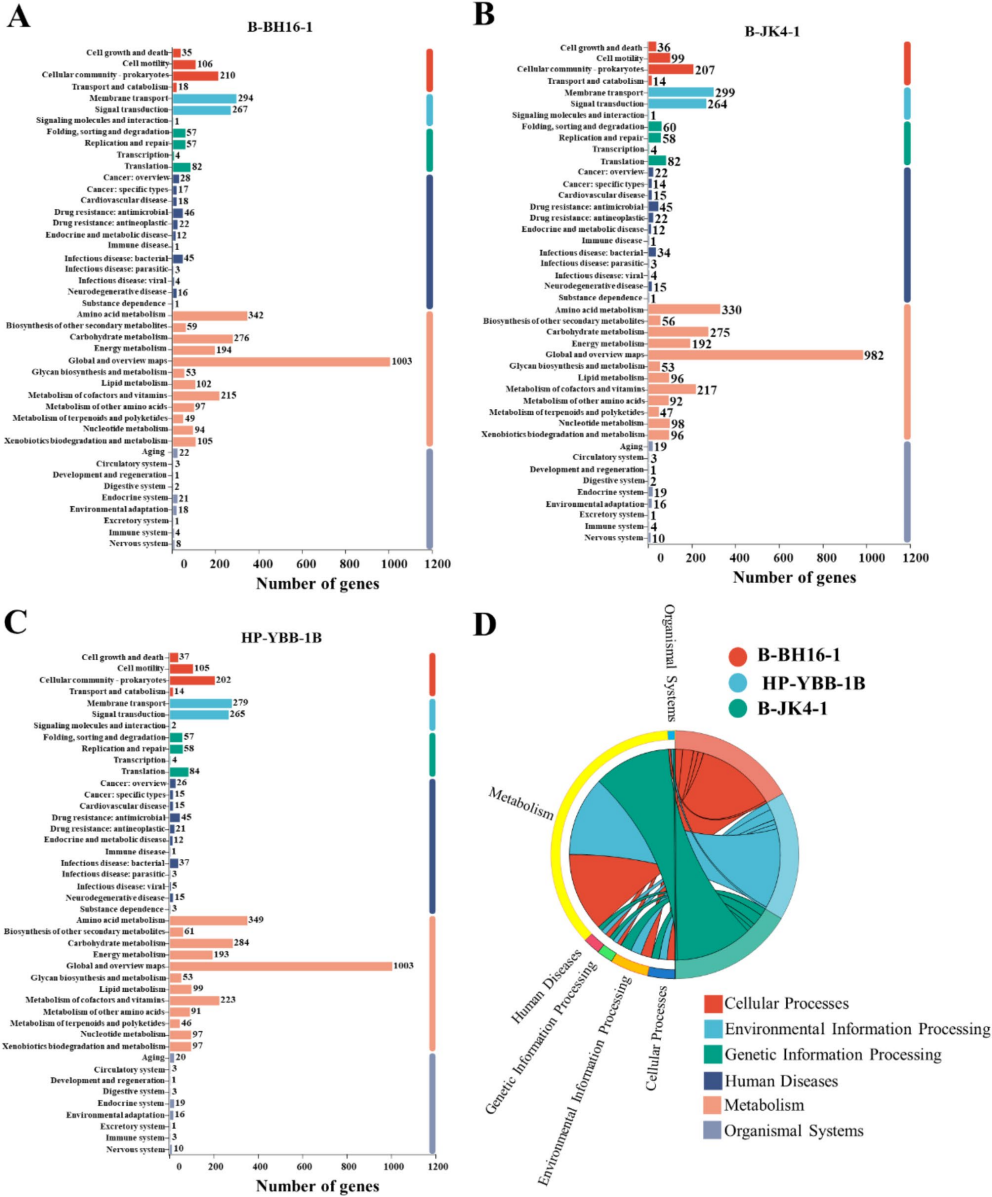
The functional annotation of the KEGG pathway for *P. palleroniana* strain B-BH16-1 (**A**), B-JK4-1 (**B**), and HP-YBB-1B (**C**). The y-axis indicates level 2 hierarchical classifications of KEGG pathways; the x-axis represents the number of genes annotated under each classification. Different colors in the columns correspond to level 1 hierarchical classifications of KEGG pathways

A gene ontology (GO) analysis showed that 4,319, 4,274, and 4,272 coding genes were annotated in strains B-BH16-1, B-JK4-1, and HP-YBB-1B, respectively (Fig. 3). In strain B-BH16-1, the annotated coding genes were categorized into molecular function (3,583), cellular component (1,963), and biological process (2,015), which were more than that in strain B-JK4-1 (3,516, 1,944, and 2,017) and HP-YBB-1B (3,539, 1,934, and 2,004). Notably, the genes of three strains mainly functional annotated to the categories including the integral component of membrane, cytoplasm, plasma membrane, DNA binding, ATP-binding transcription factor activity, and sequence-specific DNA binding, and the number of genes in each category were more than 5.21% unigenes in the genome. The KEGG analysis showed that 3,239, 3,191, and 3,170 coding genes were annotated in strains B-BH16-1, B-JK4-1, and HP-YBB-1B, respectively (Fig. 4). In strain B-BH16-1, the annotated coding genes were categorized into cellular processes (369), environmental information processing (262), genetic information processing (200), human diseases (213), metabolism (2589), and organismal systems (80). In the metabolism category, the metabolism of terpenoids and polyketides pathway, biosynthesis of other secondary metabolites pathway, and xenobiotics biodegradation and metabolism may be involved in the biosynthesis of potential antifungal secondary metabolites. In the metabolism of terpenoids and polyketides pathway, the number of genes in strains B-BH16-1, B-JK4-1, and HP-YBB-1B was 49, 47, and 46, respectively, while the number of genes in the biosynthesis of other secondary metabolites pathway was 59, 56, and 61. This result indicates these three strains have different biosynthesis abilities in second metabolites.

### Comparative phylogenetic analysis of strains B-BH16-1, B-JK4-1, and HP-YBB-1B

**Fig. 5:**
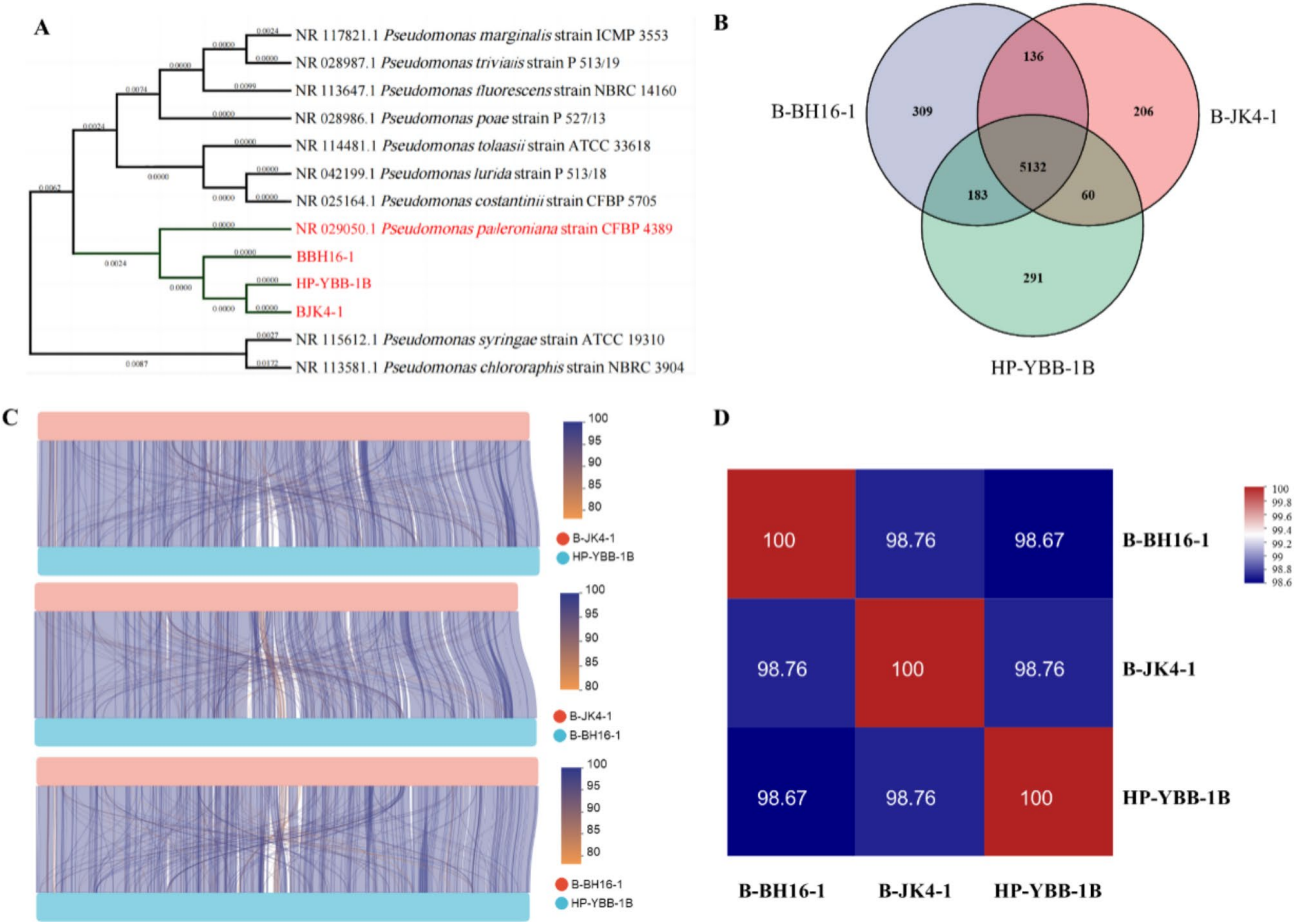
The phylogenetic analysis of *P. palleroniana* strain B-BH16-1, B-JK4-1, and HP-YBB-1B. (**A**) The phylogenetic tree of strain B-BH16-1, B-JK4-1, and HP-YBB-1B using the neighbor-joining method based on V3V4 rRNA sequences. (**B**) A VEEN map of homologous genes from these three strains. (**C**) synteny analysis of strain B-BH16-1, B-JK4-1, and HP-YBB-1B is analyzed using parallel lines. The upper and lower bars comprise color blocks representing two distinct genomes, with regions on both genomes interconnected by lines. The colors of these lines indicate the degree of collinearity. (**D**) AVI analysis among B-BH16-1, B-JK4-1, and HP-YBB-1B

To explore the phylogenetic lineage between strains B-BH16-1, B-JK4-1, and HP-YBB-1B, a comprehensive phylogenetic tree was constructed using the neighbor-joining method based on V3V4 rRNA sequences (Fig. 5A). The results showed that strains B-BH16-1, B-JK4-1, and HP-YBB-1B clustered with *Pseudomonas palleroniana* strain CFBP4389 (PP886638.1) in the same branch (Fig. 5A) Ribosomal multilocus sequence typing (rMLST) analysis based on genome sequence also showed that strains B-BH16-1, B-JK4-1, and HP-YBB-1B were most associated with *P. palleroniana* with 100% supporting value (Table S1). Type Strain Genome Server (TYGS) analysis based on genome sequence also showed that strains B-BH16-1, B-JK4-1, and HP-YBB-1B clustered with *P. palleroniana* LMG23076 with 100% identity (Figure S1). These results demonstrate that these three strains were assigned as *P. palleroniana*. In addition, the lineage between strain B-JK4-1 and B-BH16-1 or HY-YBB-1B is more closely than between strain B-BH16-1 and HP-YBB-1B. VEEN analysis showed that the shared genes between strain B-JK4-1 and B-BH16-1 or HY-YBB-1B were 5268 or 5192 genes, respectively, which is lower than that between strain B-BH16-1 and HP-YBB-1B (5315 genes) (Fig. 5B). Genome synteny analysis showed that collinearity between strain B-JK4-1 and B-BH16-1 or HY-YBB-1B was lower than between strain B-BH16-1 and HP-YBB-1B (Fig. 5B). Besides, average nucleotide identity (ANI) is a powerful approach for phylogenetic lineage assessments between bacteria. The ANI values between strain B-JK4-1 and B-BH16-1 or HY-YBB-1B were 98.76%, which is higher than that between strain B-BH16-1 and HP-YBB-1B (98.67%) (Fig. 5D). Hence, these results demonstrated that the lineage between strain B-JK4-1 and B-BH16-1 or HY-YBB-1B is more closed than between strain B-BH16-1 and HP-YBB-1B.

### Comparative analysis of cazymes in strains B-BH16-1, B-JK4-1, and HP-YBB-1B genomes

**Fig. 6:**
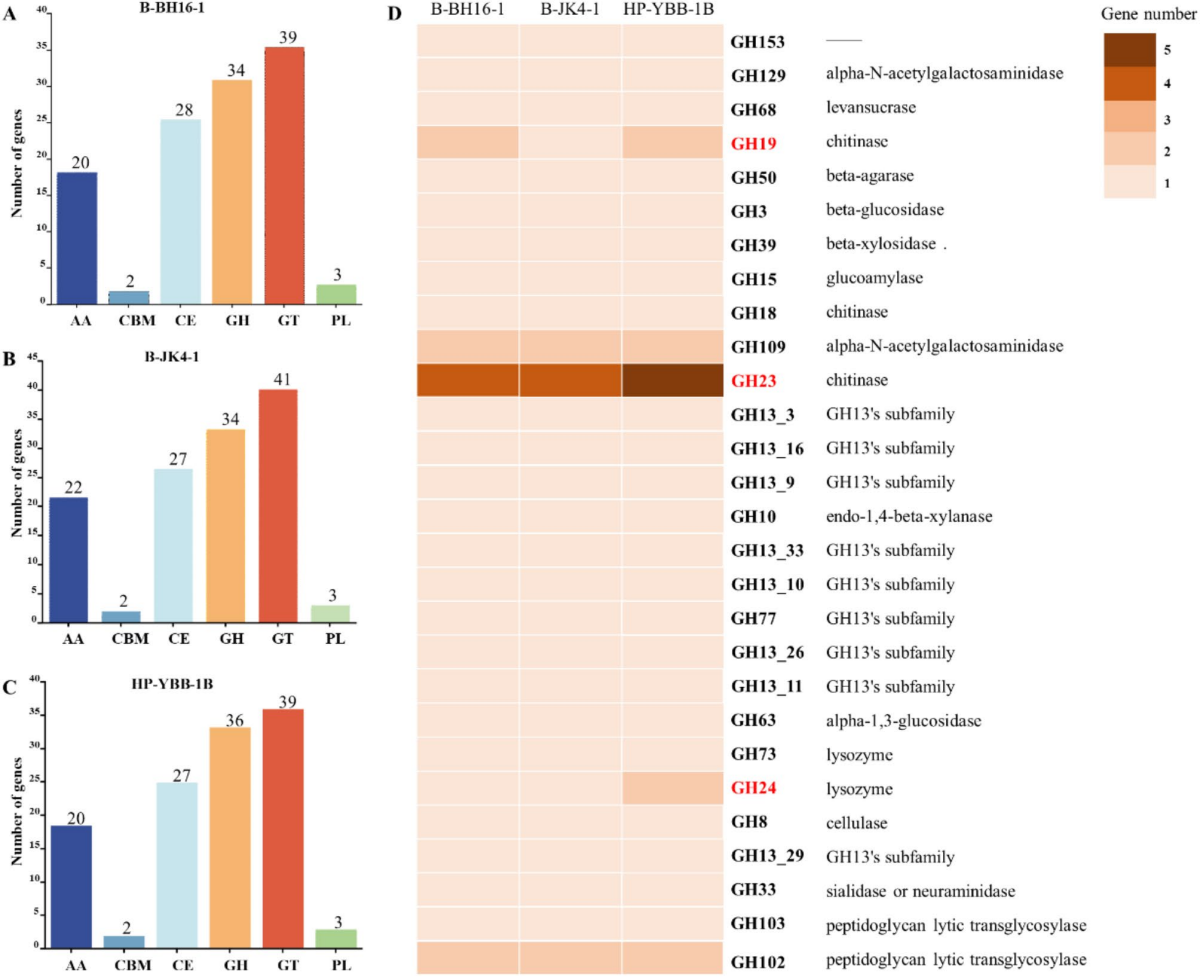
The functional characterization of the carbohydrate-active enzymes (CAZmye) in genomes of *P. palleroniana* strain B-BH16-1, B-JK4-1, and HP-YBB-1B. The number of different clusters CAZmye genes in B-BH16-1 (**A**), B-JK4-1 (**B**), and HP-YBB-1B (**C**). (**D**) The heatmap analyzes the GH cluster gene distribution in these three genes. AA: Auxiliary Activities; CBM: Carbohydrate-Binding Modules; CE: Carbohydrate Esterases; GH: Glycoside Hydrolases; GT: Glycosyl Transferases; PL: Polysaccharide Lyases

Previous research demonstrated that carbohydrate-active enzymes (CAZymes) are widely involved in antifungal defense, especially glycoside hydrolases (GH) [36, 37]. CAZyme analysis showed that 126, 129, and 127 CAZymes were identified in strains B-BH16-1, B-JK4-1, and HP-YBB-1B genomes (Fig. 6). The number of carbohydrate-binding modules (CBM) and polysaccharide lyases (PL) is 2 and 3 in these three strains, respectively. While the enzyme number of auxiliary activities (AA) and glycosyl transferases (GT) in strains B-JK4-1 (22 and 41, respectively) is higher than that in strains B-BH16-1 and HP-YBB-1B (both 20 and 39, respectively). The enzyme number of carbohydrate esterases (CE) in strain B-BH16-1 (28) is higher than that in strains B-B-JK4-1 and HP-YBB-1B (27). Intriguing, the number of glycoside hydrolases (GH) in strain HP-YBB-1B (36) is higher than that in strains B- B-JK4-1 and B-BH16-1 (34). These results indicate that these three strains can produce diverse carbohydrate-active enzymes. Notably, HP-YBB-1B produces a diverse array of GH family enzymes including chitinase (GH18, GH19, and GH23), cellulase (GH8), beta-glucosidase (GH3), alpha-glucosidase (GH63), endo-1,4-beta-xylanase (GH10), lysozyme (GH24 and GH73), levansucrase (GH68), and peptidoglycan lyases (GH102 and GH103) which exhibit potential antifungal properties (Fig. 6D). In contrast, the number of GH23 of chitinase in strain B-BH16-1 (4 genes) and B-JK4-1 (4 genes) is significantly lower than that in strain HP-YBB-1B (5 genes), while the number of GH19 of chitinase in strain B-JK4-1 (1 gene) is lower than that in strain B-BH16-1 (2 genes) and HP-YBB-1B (2 genes). Additionally, the number of GH24 of lysozyme in strain B-BH16-1 (1 gene) and B-JK4-1 (1 gene) is also significantly lower than that in strain HP-YBB-1B (2 genes). These results showed that these three strains could have different abilities to produce CAZymes in antifungal defense.

### Comparative analysis of secondary metabolite biosynthesis gene clusters in strains B-BH16-1, B-JK4-1, and HP-YBB-1B genomes

**Fig. 7:**
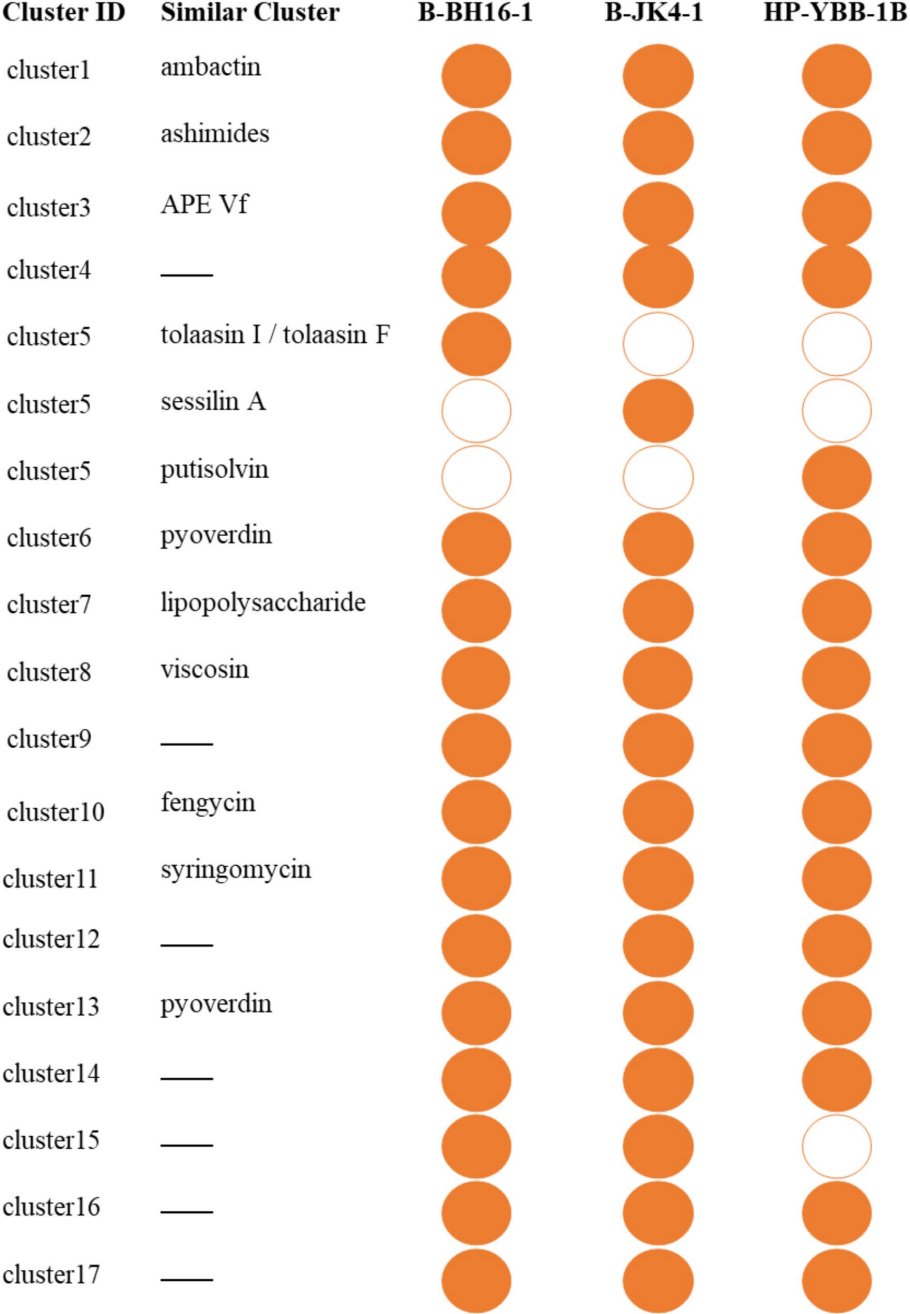
Summarizing the distribution of secondary metabolites across the three strains. The solid circle represents existing of the gene cluster for metabolite biosynthesis, while the hollow circle represents the loss of the gene cluster for metabolite biosynthesis

The secondary metabolites produced by bacteria had strong antifungal activities [38]. The antiSMASH analysis based on MiBIG database revealed that strains B-BH16-1, B-JK4-1, and HP-YBB-1B collectively identified at 17, 16, and 16 clusters associated with the biosynthesis of secondary metabolites, including hypothetical protein (tolaasin I / tolaasin F, sessilin A, and putisolvin), lipopeptides (fengycin, viscosin, and syringomycin), siderophores (pyoverdin and ambactin), polypeptides (ashimides), and aromatic polyenes (APE Vf) (Fig. 7, S2 and Tables 3, 4 and 5). Among them, only cluster 5 has more than 70% similarity against MiBIG database of known secondary metabolites, while 9 clusters (clusters 3, 8, 11, 1, 10, 6, 13, 2, and 7) have 5 ~ 45% similarity only. Intriguing, there are 7, 7, and 6 clusters in strains B-BH16-1, B-JK4-1, and HP-YBB-1B with no similarity against the MiBIG database of known secondary metabolites, respectively. These results mean that these predicted compounds with low or no similarity may be novel metabolites.

**Table 3:** Analysis of antibiotic and secondary metabolite biosynthesis gene clusters in strain B-BH16-1

Cluster ID	Type	Start	End	Similar Cluster	Similarity (%)	Gene No.
cluster5	MULTISPECIES: hypothetical protein	2,186,556	2,243,137	tolaasin I / tolaasin F	80	23
cluster3	arylpolyene	518,673	562,249	APE Vf	45	40
cluster8	RHS repeat-associated core domain-containing protein	3,901,957	3,964,247	viscosin	43	36
cluster11	-	4,231,377	4,277,156	syringomycin	35	40
cluster1	MULTISPECIES: DUF2388 domain-containing protein	123,567	152,369	ambactin	25	21
cluster10	betalactone	4,101,625	4,124,707	fengycin	13	19
cluster6	ABCtransporter permease subunit	2,452,054	2,516,288	pyoverdin	10	35
cluster13	NRPS	4,522,348	4,575,236	pyoverdin	9	39
cluster2	EAL domain-containing protein	267,398	317,187	ashimides	8	44
cluster7	thiopeptide	3,590,411	3,616,258	lipopolysaccharide	5	26
cluster4	Hypothetical protein	1,534,813	1,543,517	-	-	12
cluster9	Hypothetical protein	4,048,541	4,058,369	-	-	14
cluster12	NAGGN	4,470,491	4,485,209	-	-	11
cluster14	siderophore	4,660,925	4,672,854	-	-	11
cluster15	Fatty acid desaturase	5,124,943	5,146,330	-	-	26
cluster16	TfuA-related	5,235,968	5,257,855	-	-	31
cluster17	bacteriocin	6,601,521	6,612,367	-	-	10

Note: “-” indicates the gene cluster in the B-BH16-1 genome that has not been matched in the MiBIG database

**Table 4:** Analysis of antibiotic and secondary metabolite biosynthesis gene clusters in strain B-JK4-1

Cluster ID	Type	Start	End	Similar Cluster	Similarity (%)	Gene No.
cluster5	Transketolase	2,217,905	2,315,799	sessilin A	100	37
cluster3	arylpolyene	508,691	552,267	APE Vf	45	40
cluster8	RHS repeat-associated core domain-containing protein	3,672,098	3,735,358	viscosin	43	34
cluster11	-	4,012,179	4,057,593	syringomycin	35	36
cluster1	MULTISPECIES: DUF2388 domain-containingprotein	123,659	152,313	ambactin	25	21
cluster10	betalactone	3,881,013	3,904,094	fengycin	13	19
cluster6	ABC transporter permease subunit	2,486,586	2,551,902	pyoverdin	10	36
cluster13	NRPS	4,357,535	4,410,423	pyoverdin	10	39
cluster2	EAL domain-containing protein	261,406	311,237	ashimides	8	45
cluster7	thiopeptide	3,361,369	3,387,306	lipopolysaccharide	5	28
cluster4	Hypothetical protein	1,526,930	1,535,633	-	-	12
cluster9	MULTISPECIES: hypothetical protein	3,827,898	3,837,725	-	-	14
cluster12	NAGGN	4,305,742	4,320,460	-	-	11
cluster14	siderophore	4,496,304	4,508,233	-	-	11
cluster15	Fatty acid desaturase	-	-	-	-	-
cluster16	TfuA-related	4,895,185	4,917,073	-	-	25
cluster17	bacteriocin	6,357,100	6,367,946	-	-	10

Note “-” indicates the gene cluster in the B-JK4-1 genome that has not been matched in the MiBIG database

**Table 5:** Analysis of antibiotic and secondary metabolite biosynthesis gene clusters in strain HP-YBB-1B

Cluster ID	Type	Start	End	Similar Cluster	Similarity (%)	Gene No.
cluster5	MULTISPECIES: hypothetical protein	2,188,192	2,283,762	putisolvin	100	39
cluster3	arylpolyene	502,116	545,692	APE Vf	45	42
cluster8	RHS repeat-associated core domain-containing protein	3,849,171	3,911,487	viscosin	43	34
cluster11	-	4,182,850	4,228,515	syringomycin	35	40
cluster1	MULTISPECIES: DUF2388 domain-containing protein	107,415	136,069	ambactin	25	21
cluster10	betalactone	4,048,500	4,071,582	fengycin	13	19
cluster6	ABC transporter permease subunit	2,434,270	2,499,673	pyoverdin	10	36
cluster13	NRPS	4,571,859	4,624,747	pyoverdin	9	39
cluster2	EAL domain-containing protein	257,275	307,129	ashimides	8	47
cluster7	thiopeptide	3,543,310	3,569,156	lipopolysaccharide	5	25
cluster4	Hypothetical protein	1,503,900	1,512,603	-	-	12
cluster9	Hypothetical protein	3,995,676	4,005,502	-	-	14
cluster12	NAGGN	4,520,119	4,534,837	-	-	10
cluster14	siderophore	4,708,904	4,720,833	-	-	11
cluster15	Fatty acid desaturase	-	-	-	-	-
cluster16	TfuA-related	5,131,849	5,153,737	-	-	25
cluster17	bacteriocin	6,491,457	6,502,303	-	-	10

Note “-” indicates the gene cluster in the HP-YBB-1B genome that has not been matched in the MiBIG database

**Fig. 8:**
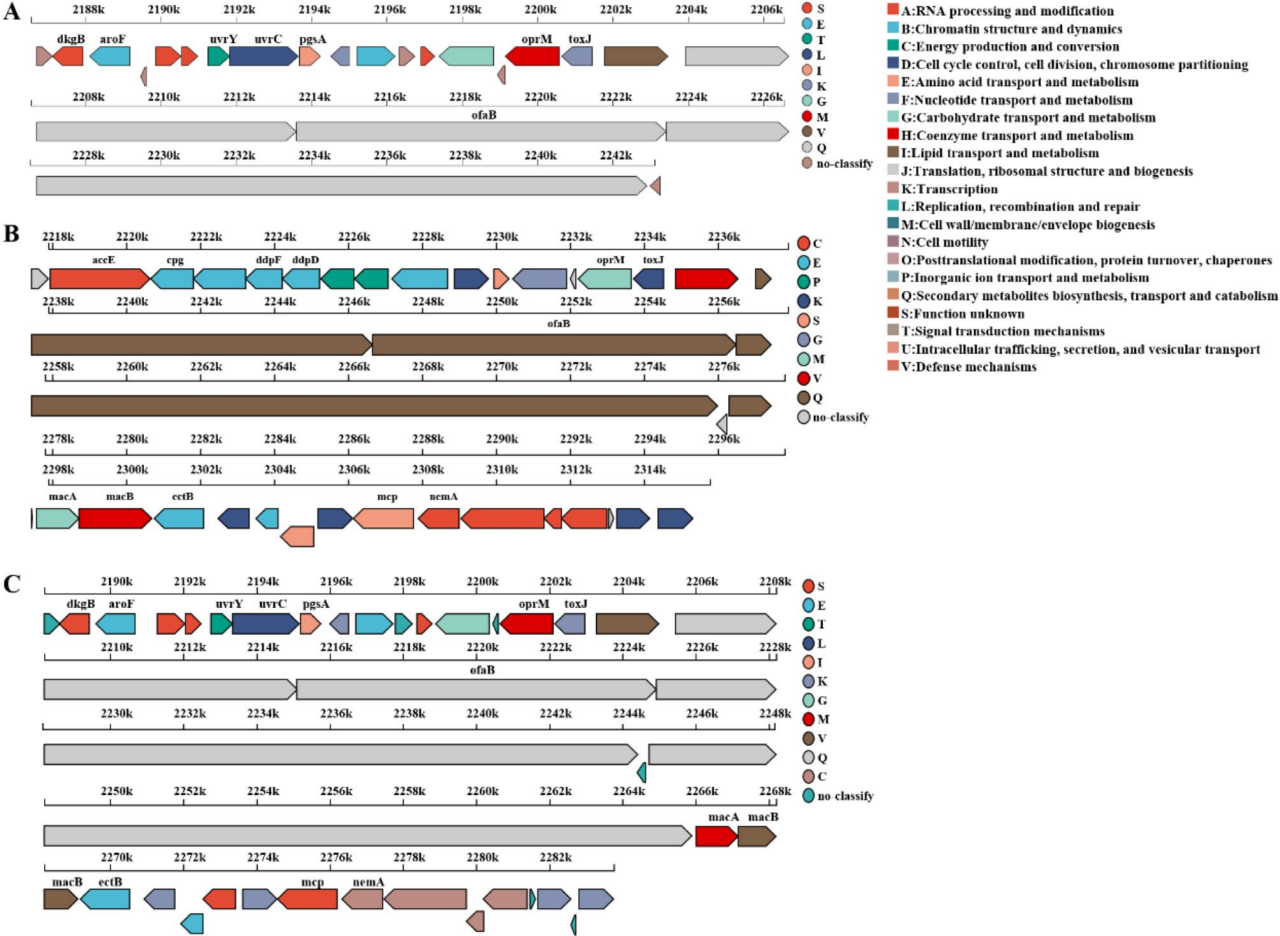
Linear gene cluster diagram of the biosynthesis gene clusters. (**A**) The gene cluster of tolaasin I/tolaasin F in strain B-BH16-1. (**B**) The gene cluster of sessilin A in strain B-JK4-1. (**C**) The gene cluster of putisolvin in strain HP-YBB-1B. The length and orientation of arrows indicate gene length and coding direction, respectively, while arrow color signifies COG classification

**Fig. 9:**
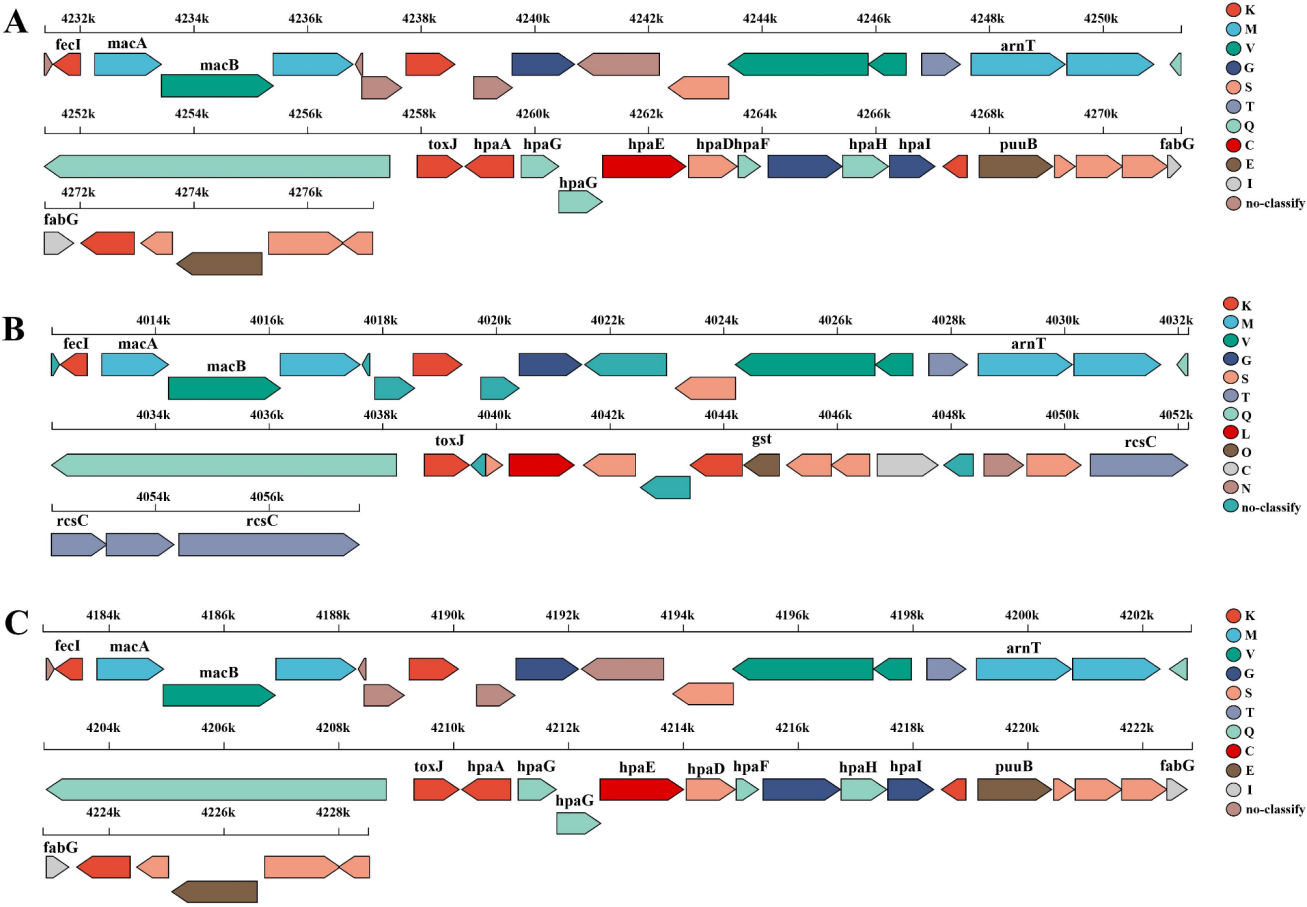
Linear gene cluster diagram of the biosynthesis gene clusters of syringomycin in *P. palleroniana* strain B-BH16-1 (**A**), B-JK4-1 (**B**), and HP-YBB-1B (**C**). These diagrams depict all predicted genes within the clusters, with distinct colors denoting their respective COG classifications. Each color signifies a specific function, as the COG analysis interface outlines. Genes that do not possess a COG annotation are illustrated in gray

Notably, the number of genes in the same secondary metabolite biosynthesis cluster differs among these three strains. Specifically, in cluster 5, the predicted compounds are significantly different among these three strains: tolaasin I / tolaasin F (23 genes), sessilin A (37 genes), and putisolvin (39 genes) is the member of cluster 5 in strains B-BH16-1, B-JK4-1, and HP-YBB-1B, respectively (Fig. 8). In addition, cluster 15 (fatty acid desaturase) exists in strain B-BH16-1 while lost in strains B-JK4-1 and HP-YBB-1B (Fig. 7). Furthermore, in the polypeptides syringomycin biosynthesis gene cluster, the gene number is 40, 36, and 40 in strains B-BH16-1, B-JK4-1, and HP-YBB-1B, respectively (Fig. 9). Strains B-BH16-1 and HP-YBB-1B exhibited consistent coding genes in syringomycin biosynthesis gene clusters, while strain B-JK4-1 displayed distinct coding genes. We found that the differing genes were associated with biosynthesis enzymes (hpaA, hpaG, hpaE, hpaF, hpaH, and puuB), regulatory factors (hpaI), as well as hypothetical proteins (hpaG and hpaD). In comparison, these three strains shared identical gst genes (proton conductive membrane transporter protein), rcsC genes (signal peptidase), and rcsC related to tRNA synthetase (Fig. 9). The pathogen-driven three *P. palleroniana* strains have divergent gene clusters for antifungal metabolites biosynthesis, which may result in a different antifungal spectrum against the dominant pathogens of *P. heterophylla*.

## Discussions

Multiple pathogens can result in various serious diseases (i.e., root rot, Fusarium wilt, and leaf spot) in *P. hererophylla* [7, 27, 39], thereby unearthing broad-spectrum antifungal bacteria resources is vital for preventing and managing the primary devastation diseases in *P. hererophylla*. Herein, we obtained three strains (B-BH16-1, B-JK4-1, and HP-YBB-1B) with broad-spectrum antifungal activity against the eleven dominant pathogens in *P. hererophylla* and their inhibitory spectrum is different (Fig. 1). Phylogenetic analysis based on 16 S rRNA (Fig. 5) and rMLST and TYGS species designation analysis based on genome sequence (Figure S1 and Table S2) showed that strains B-BH16-1, B-JK4-1, and HP-YBB-1B were identified as *P. palleroniana*. Additionally, synteny and ANI analysis showed that the lineage between strain B-JK4-1 with B-BH16-1 or HY-YBB-1B was closer than that between strain B-BH16-1 with HP-YBB-1B (Fig. 5C and D). Previous research found that P. aeruginosa, P. fluorescens, and P. putida can synthesize antifungal metabolites that directly inhibit pathogen growth and repress disease [18–23]. These three *P. palleroniana* strains were the new species that owe the antifungal ability to control plant disease [1]. Therefore, these strains provide various new biocontrol resources for constructing synthetic community agents to manage multiple diseases in *P. hererophylla* in the future.

The safety application of antifungal bacteria is a significant concern. Our previous research proved that P. palleroniana have antifungal activity against P. heterophylla pathogens [1]. However, other previous research has found that *P. palleroniana* has been identified as a phytopathogen infecting *Oryza sativa* [40] and causing soft rot in potato tubers [41]. Additionally, we have identified certain genes categorized under human diseases in these three *P. palleroniana* strains (Fig. 4). Therefore, the applicability and potential risks of these three strains should be clarified.

The secondary metabolites of bacteria are the main antifungal compounds. The inhibitory activity of bacteria is closely associated with the metabolite types and production ability of antifungal compounds [42]. The comparative genome of strains B-BH16-1, B-JK4-1, and HP-YBB-1B showed that the secondary metabolite biosynthesis genes among these three *P. palleroniana* strains exhibit marked differences (Fig. 7, S2 and Tables 3, 4 and 5). Among these three strains, there are 17, 17, and 16 clusters in strains B-BH16-1, B-JK4-1, and HP-YBB-1B, respectively. Cluster 15 (fatty acid desaturase) exists in strain B-BH16-1 while lost in strains B-JK4-1 and HP-YBB-1B (Fig. 7). Among these predicted clusters, only cluster 5 has more than 70% similarity, while nine predicted clusters have low similarity, and seven predicted clusters have no similarity against the MiBIG database (Tables 3, 4 and 5). In cluster 5, the predicted compounds are significantly different among these three strains, B-BH16-1, B-JK4-1, and HP-YBB-1 produced tolaasin I/tolaasin F (23 genes), sessilin A (37 genes), and putisolvin (39 genes), respectively (Fig. 8). Previous studies showed these three compounds have different antifungal activity and mode action. The tolaasin I/ tolaasin F, an antimicrobial lipopeptide predicted in strain B-BH16-1, can combine with the fungal cell membrane to form pores, which causes an increase in membrane permeability and this membrane damage results in the loss of crucial substances (such as ions, nucleic acids, and proteins) within cells, eventually leading to cell death [43, 44]. It is found that tolaasin I/ tolaasin F can be produced by *P. tolaasii*, demonstrating antifungal properties and exhibiting inhibitory effects against diverse pathogens [45, 46]. However, this is the first record of tolaasin I/ tolaasin F production by *P. palleroniana*. The cyclic lipopeptide sessilin A predicted in strain B-JK4-1 offers promising applications in drug development, particularly in anti-tumor, anti-inflammatory, and antimicrobial activities [47]. It has been demonstrated that sessilin plays a vital role in suppressing tomato wilt disease, soybean root rot disease, and sweet potato yellow rot disease [48, 49]. Putisolvin, a polyketide predicted in strain HP-YBB-1B, has antagonistic activity against various pathogens and generally plays an antifungal role by destroying cell membranes and inhibiting DNA synthesis. Additionally, it can function as a biological surfactant that modulates bacterial quorum sensing and biofilm formation [50, 51].

Polypeptides such as ambactin, viscosin, fengycin, and syringomycin were also predicted and annotated in these three isolate genomes, which have been identified with strong antifungal activity against plant pathogens [50, 52, 53]. However, a comparative analysis of their biosynthetic gene clusters found that their similarity was below 50% in these three strains. Furthermore, strains B-BH16-1, B-JK4-1, and HP-YBB-1B predicted 7, 7, and 6 secondary metabolite biosynthetic gene clusters, respectively, while these clusters did not match known metabolites in the MiBIG database. These findings imply that these predicted secondary metabolite biosynthesis gene clusters of B-BH16-1, B-JK4-1, and HP-YBB-1B might synthesize other unreported similar or new substances in addition to the aforementioned known compounds in the database. Therefore, these strains may possess diverse metabolites, which have been extensively researched for their potential antifungal activities.

The variations in the gene coding of identical secondary metabolite biosynthesis gene clusters may arise from several factors. Within the same species, different individuals or strains can undergo gene mutations, insertions, or deletions that contribute to genetic diversity. A hallmark of prokaryotic genome evolution is the frequent acquisition of genes through horizontal gene transfer (HGT), which can result in gene rearrangement, transposition, or further instances of horizontal gene transfer [54]. These processes may lead to structural differences within the same secondary metabolite biosynthesis gene cluster across various strains, even though the final synthesized products remain consistent. Identical metabolites may comprise distinct coding genes, and their expression regulation mechanisms might differ—such as promoter strength, epigenetic modifications, and transcription factor binding sites—all contributing to variations in how these metabolites are synthesized among individuals [55, 56]. Microorganisms exhibit substantial genetic variation that results in significant discrepancies in metabolite synthesis coding [39, 57]. Previous studies revealed the evolutionary mechanism that controls the structural novelty during the biosynthesis of metabolites [58]. However, over 50% of strains do not express these metabolites under current laboratory conditions and are thus classified as “silent,” “latent,” or “orphan” gene clusters [59], indicating that differences in the coding for identical secondary metabolite biosynthesis gene clusters could also stem from these factors.

Most pathogen cell walls comprise chitin and beta-glucan [60, 61]. Chitinase catalyzes the hydrolysis of chitin to yield N-acetylglucosamine, which impairs the structural integrity of fungal cell walls, thereby hindering the germination of spores and the growth of mycelium in some fungi [38, 62]. Similarly, beta-glucan can also disrupt the structural integrity of the fungal cell wall [63]. B-BH16-1, B-JK4-1 and HP-YBB-1B genomes contain 127, 129, and 127 CAZymes coding genes. These CAZymes encompass a substantial number of genes related to cell walls degrading enzymes, such as beta-glucanase, xylan 1,4-xylosidase, glucan 1,3-beta-glucosidase, glucan 1,4-beta-glucosidase, isopentose-producing oligoxyglucan hydrolase, and chitinase. The CAZymes family also encompasses a considerable number of genes related to lysozyme. It is acknowledged that lysozyme functions by inducing programmed cell death, which entails the loss of mitochondrial membrane potential, phosphatidylserine exposure on the outer leaflets of the cell membrane, chromatin condensation, and DNA fragmentation [53]. Therefore, these three strains are hypothesized to degrade pathogen cell walls and induce programmed cell death as part of their antifungal mechanisms.

## Electronic supplementary material

Below is the link to the electronic supplementary material.

Supplementary Material 1

## Data Availability

The raw data used in this study are publicly available at NCBI under the project accession PRJNA1203432. The biosample accessions were SAMN45996118, SAMN45996119, and SAMN45996120.
