# Supplementary material for: The comparative genomic analysis provides insight into the divergent inhibitory activity metabolites in pathogen-driven three Pseudomonas palleroniana strains against primary pathogens of Pseudostellaria heterophylla
Source: BMC Genomics. 2025 Apr 2;26:332. doi: 10.1186/s12864-025-11527-8 (PMC11963402; doi:10.1186/s12864-025-11527-8)
Supplement: Supplementary file 1 — Supplementary Material 1 [file 12864_2025_11527_MOESM1_ESM.docx]

**The comparative genomic analysis provides insight into the divergent inhibitory activity metabolites in pathogen-driven three *Pseudomonas palleroniana* strains against primary pathogens of *Pseudostellaria heterophylla***

Chunfeng Huang^1,#^, Xiaoai Wang^1,#^, Yanping Gao^1^, Xue Jiang^2^, Lingling Wang^1^, Xiaohong Ou^1^, Yanhong Wang^3^, Tao Zhou^1,†^, and Qing-Song Yuan^1,3,†^

^1^ Resource Institute for Chinese & Ethnic Materia Medica, Guizhou University of Traditional Chinese Medicine, Guiyang 550025, China; Guizhou Key Laboratory for Germplasm Innovation and Resource-Efficient Utilization of Dao-di Herbs. 1157680721@qq.com (CFH);  [1925426266@qq.com](mailto:2110499772@qq.com) (XAW); gaoyanping087@gzy.edu.cn (YPG); 1214144081@qq.com (LLW); yanhong824@hotmail.com (YHW); [ogh1986@163.com](mailto:ogh1986@163.com) (XHO); [taozhou88@163.com](mailto:taozhou88@163.com) (TZ); [yqs198609031006@126.com](mailto:yqs198609031006@126.com) (QSY)

^2^ School of Food and Biological Engineering, Jiangsu University, Zhenjiang, 212013, China. [1017036604@qq.com](mailto:1017036604@qq.com) (XJ);

^3^ National Resource Center for Chinese Materia Medica, China Academy of Chinese Medical Sciences, Beijing 100700; China State Key Laboratory for Quality Ensurance and Sustainable Use of Dao-di Herbs. [glp01@126.com](mailto:glp01@126.com) (LPG);

# Authors have equal work in this paper.

†Corresponding authors

Qing-Song Yuan

Guizhou University of Traditional Chinese Medicine; National Resource Center for Chinese Meteria Medica; State Key Laboratory for Quality Ensurance and Sustainable Use of Dao-di Herbs

Email: [yqs198609031006@126.com](mailto:yqs198609031006@126.com), [yuanqingsong006@gzy.edu.cn](mailto:Yuanqingsong006@gzy.edu.cn);

Tao Zhou

Guizhou University of Traditional Chinese Medicine

Email: taozhou88@163.com, [zhoutao700@gyctcm.edu.cn](mailto:Yuanqingsong006@gzy.edu.cn).


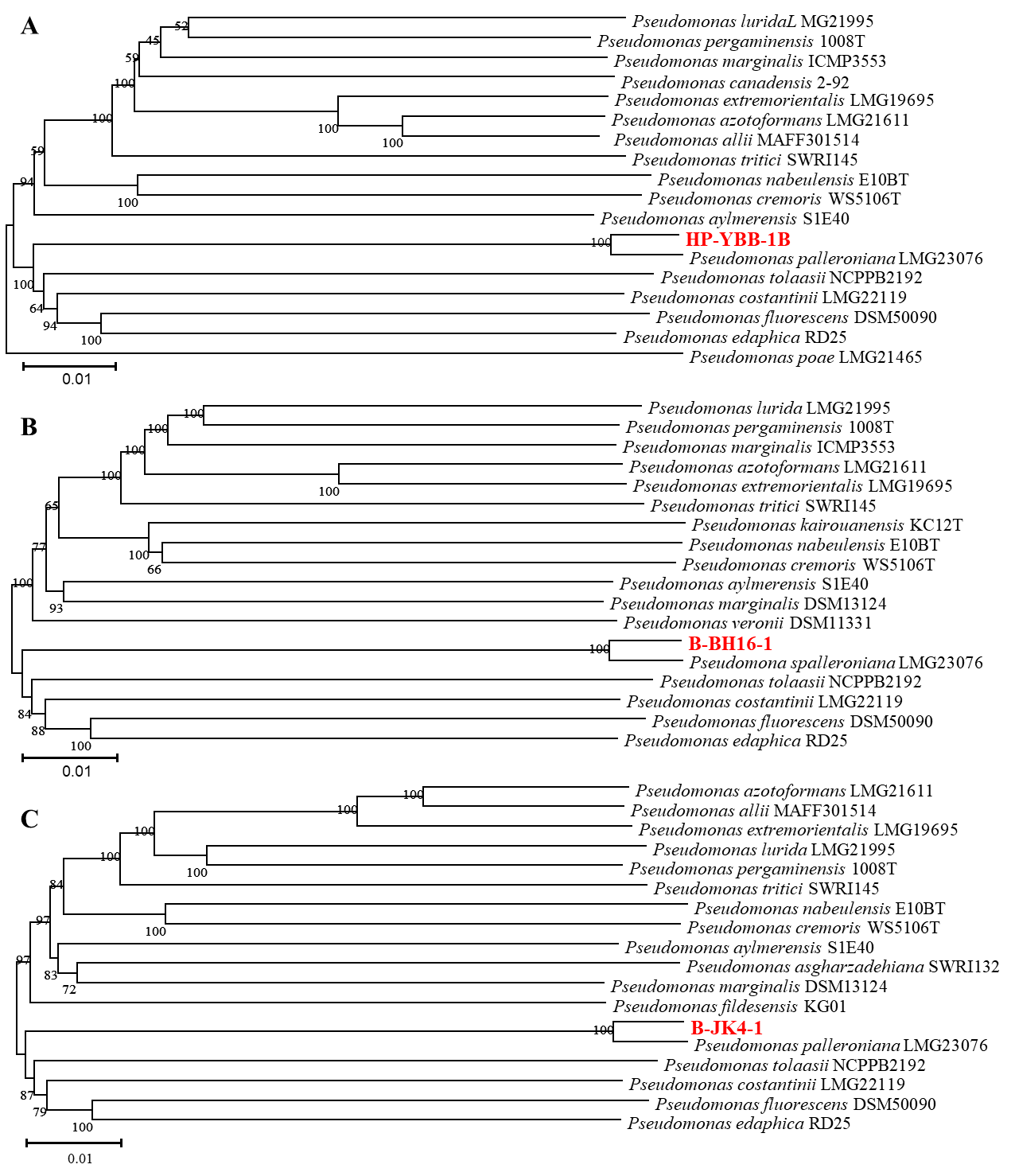


**Figure S1** **TYGS analysis for the species designation of strains HP-YBB-1B (A), B-BH16-1 (B), and B-JK4-1(C) based on genome sequence.**


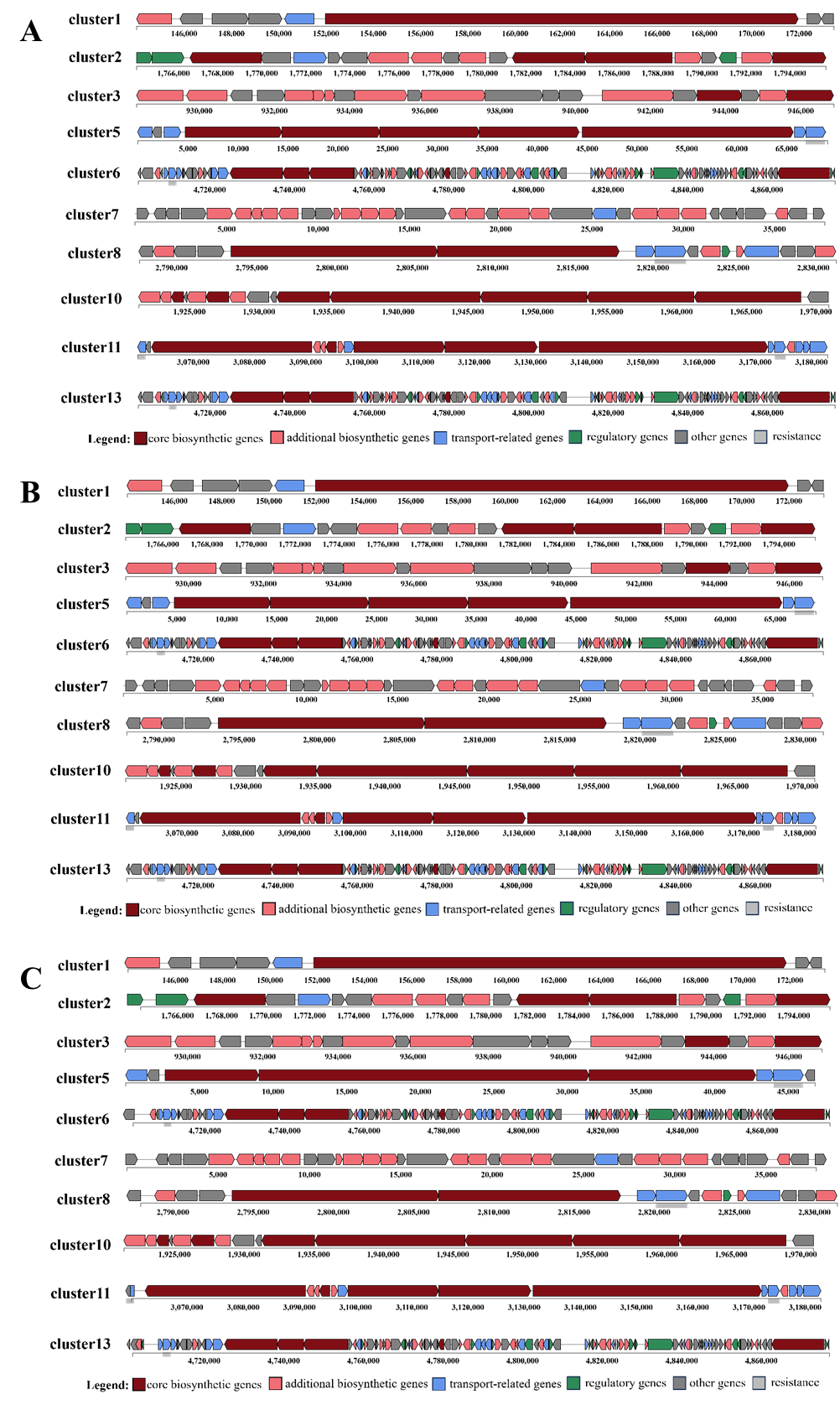


**Figure S2** **Prediction of gene clusters associated with the biosynthesis of secondary metabolites using the AntiSMASH online platform.** Different colors denote distinct types of genes, where A represents B-BH16-1, B denotes B-JK4-1, and C corresponds to HP-YBB-1B.

**Table S1** **rMLST analysis for the species designation of strains B-BH16-1, B-JK4-1, and HP-YBB-1B based on genome sequence.**

| **Strains** | **Locus** | **Allele** | **Length** | **Start position** | **End position** | **Linked data values** | **Flags** |
| --- | --- | --- | --- | --- | --- | --- | --- |
| **B-JK4-1** | BACT000001 | 32226 | 1686 | 1823498 | 1825183 | **species:** *Pseudomonas palleroniana*[n=1] |  |
| **B-JK4-1** | BACT000032 | 25948 | 636 | 5634435 | 5635070 | **species:** *Pseudomonas palleroniana*[n=1] |  |
| **B-JK4-1** | BACT000002 | 14458 | 738 | 1451423 | 1452160 | **species:** *Pseudomonas palleroniana* [n=6]; *Pseudomonas sp.* [n=2] |  |
| **B-JK4-1** | BACT000004 | 29313 | 621 | 5623214 | 5623834 | **species:***Pseudomonas palleroniana* [n=2]; *Pseudomonas sp.*[n=2] |  |
| **B-JK4-1** | BACT000007 | 11457 | 471 | 5638981 | 5639451 | **species:***Pseudomonas palleroniana* [n=7] ; *Pseudomonas sp.*[n=2] |  |
| **B-JK4-1** | BACT000009 | 15556 | 393 | 1007820 | 1008212 | **species:***Pseudomonas palleroniana* [n=7] ; *Pseudomonas sp.*[n=1] |  |
| **B-JK4-1** | BACT000016 | 11007 | 252 | 5104887 | 5105138 | **species:***Pseudomonas palleroniana* [n=8] ; *Pseudomonas sp.*[n=2] |  |
| **B-JK4-1** | BACT000020 | 9728 | 279 | 923212 | 923490 | **species:***Pseudomonas palleroniana* [n=5] ; *Pseudomonas sp.*[n=2] |  |
| **B-JK4-1** | BACT000030 | 15571 | 696 | 5649860 | 5650555 | **species:***Pseudomonas palleroniana* [n=3] ; *Pseudomonas sp.*[n=1] |  |
| **B-JK4-1** | BACT000033 | 13191 | 603 | 5633820 | 5634422 | **species:***Pseudomonas palleroniana* [n=3] ; *Pseudomonas sp.*[n=1] |  |
| **B-JK4-1** | BACT000034 | 11507 | 540 | 5629183 | 5629722 | **species:***Pseudomonas palleroniana* [n=7] ; *Pseudomonas sp.*[n=2] |  |
| **B-JK4-1** | BACT000060 | 11301 | 219 | 505956 | 506174 | **species:***Pseudomonas palleroniana* [n=5]; *Pseudomonas sp.*[n=2] |  |
| **B-BH16-1** | BACT000039 | 11276 | 501 | 5999808 | 6000308 | **species:** *Pseudomonas palleroniana* [n=6] |  |
| **B-BH16-1** | BACT000020 | 81858 | 279 | 935445 | 935723 | **species:** *Pseudomonas palleroniana* [n=1] |  |
| **B-BH16-1** | BACT000033 | 94549 | 603 | 5984468 | 5985070 | **species:** *Pseudomonas palleroniana* [n=2] |  |
| **B-BH16-1** | BACT000035 | 27661 | 534 | 5978365 | 5978898 | **species:** *Pseudomonas palleroniana* [n=1] |  |
| **B-BH16-1** | BACT000005 | 12424 | 501 | 5977500 | 5978000 | **species:** *Pseudomonas palleroniana* [n=4]; *Pseudomonas sp.* [n=1] |  |
| **B-BH16-1** | BACT000008 | 10808 | 393 | 5978911 | 5979303 | **species:** *Pseudomonas palleroniana* [n=7]; *Pseudomonas sp.* [n=2] |  |
| **B-BH16-1** | BACT000009 | 12017 | 393 | 1020047 | 1020439 | **species:** *Pseudomonas palleroniana* [n=1]; *Pseudomonas sp.* [n=1] |  |
| **B-BH16-1** | BACT000011 | 13648 | 390 | 5974501 | 5974890 | **species:** *Pseudomonas palleroniana* [n=7]; *Pseudomonas sp.* [n=2] |  |
| **B-BH16-1** | BACT000012 | 11151 | 372 | 5990218 | 5990589 | **species:** *Pseudomonas palleroniana* [n=7]; *Pseudomonas sp.* [n=2] |  |
| **B-BH16-1** | BACT000016 | 11007 | 252 | 5445021 | 5445272 | **species:** *Pseudomonas palleroniana* [n=8]; *Pseudomonas sp.* [n=2] |  |
| **B-BH16-1** | BACT000030 | 15571 | 696 | 6000508 | 6001203 | **species:** *Pseudomonas palleroniana* [n=3]; *Pseudomonas sp.* [n=1] |  |
| **B-BH16-1** | BACT000031 | 13705 | 825 | 5983333 | 5984157 | **species:** *Pseudomonas palleroniana* [n=8]; *Pseudomonas sp.* [n=2] |  |
| **B-BH16-1** | BACT000032 | 13165 | 636 | 5985083 | 5985718 | **species:** *Pseudomonas palleroniana* [n=3];*Pseudomonas sp.* [n=1] |  |
| **B-BH16-1** | BACT000036 | 10481 | 366 | 5999364 | 5999729 | **species:** *Pseudomonas palleroniana* [n=7]; *Pseudomonas sp.* [n=2] |  |
| **B-BH16-1** | BACT000038 | 13228 | 447 | 670639 | 671085 | **species:** *Pseudomonas palleroniana* [n=5]; *Pseudomonas sp.* [n=1] |  |
| **B-BH16-1** | BACT000040 | 15618 | 432 | 6001203 | 6001634 | **species:** *Pseudomonas palleroniana* [n=4]; *Pseudomonas sp.* [n=1] |  |
| **B-BH16-1** | BACT000042 | 10574 | 429 | 1019604 | 1020032 | **species:** *Pseudomonas palleroniana* [n=8]; *Pseudomonas sp.* [n=1] |  |
| **B-BH16-1** | BACT000044 | 25420 | 438 | 5976880 | 5977317 | **species:** *Pseudomonas palleroniana* [n=4]; *Pseudomonas sp.* [n=1] |  |
| **B-BH16-1** | BACT000045 | 11503 | 414 | 5981571 | 5981984 | **species:** *Pseudomonas palleroniana* [n=7]; *Pseudomonas sp.* [n=1] |  |
| **B-BH16-1** | BACT000049 | 11211 | 357 | 4334476 | 4334832 | **species:** *Pseudomonas palleroniana* [n=6]; *Pseudomonas sp.* [n=2] |  |
| **B-BH16-1** | BACT000053 | 10378 | 315 | 5980393 | 5980707 | **species:** *Pseudomonas palleroniana* [n=7]; *Pseudomonas sp.* [n=1] |  |
| **B-BH16-1** | BACT000056 | 11027 | 258 | 5594465 | 5594722 | **species:** *Pseudomonas palleroniana* [n=8]; *Pseudomonas sp.* [n=2] |  |
| **B-BH16-1** | BACT000060 | 11301 | 219 | 515939 | 516157 | **species:** *Pseudomonas palleroniana* [n=5]; *Pseudomonas sp.* [n=2] |  |
| **B-BH16-1** | BACT000061 | 8800 | 183 | 4989535 | 4989717 | **species:** *Pseudomonas palleroniana* [n=4]; *Pseudomonas sp.* [n=1] |  |
| **B-BH16-1** | BACT000063 | 8241 | 135 | 6655780 | 6655914 | **species:** *Pseudomonas palleroniana* [n=8]; *Pseudomonas sp.* [n=7] |  |
| **B-BH16-1** | BACT000006 | 11143 | 423 | 669009 | 669431 | **species:** *Pseudomonas palleroniana* [n=8]; *Pseudomonas sp.*[n=3] |  |
| **B-BH16-1** | BACT000043 | 9919 | 369 | 5980719 | 5981087 | **species:** *Pseudomonas palleroniana* [n=7]; *Pseudomonas mucidolens* [n=1] |  |
| **B-BH16-1** | BACT000047 | 10869 | 351 | 5978004 | 5978354 | **species:** *Pseudomonas sp.*[n=11]; *Pseudomonas palleroniana* [n=8] |  |
| **B-BH16-1** | BACT000051 | 9458 | 333 | 5982697 | 5983029 | **species:** *Pseudomonas sp.* [n=11]; *Pseudomonas palleroniana* [n=8] |  |
| **HP-YBB-1B** | BACT000038 | 27771 | 447 | 654037 | 654483 | **species:** *Pseudomonas palleroniana* [n=2] |  |
| **HP-YBB-1B** | BACT000003 | 12855 | 687 | 5870106 | 5870792 | **species:** *Pseudomonas palleroniana* [n=7]; *Pseudomonas sp.* [n=2] |  |
| **HP-YBB-1B** | BACT000016 | 11007 | 252 | 5341257 | 5341508 | **species:** *Pseudomonas palleroniana* [n=8]; *Pseudomonas sp.* [n=2] |  |
| **HP-YBB-1B** | BACT000040 | 26649 | 432 | 5889312 | 5889743 | **species:** *Pseudomonas palleroniana* [n=1]; *Pseudomonas sp.* [n=1] |  |
| **HP-YBB-1B** | BACT000060 | 11301 | 219 | 498935 | 499153 | **species:** *Pseudomonas palleroniana* [n=5]; *Pseudomonas sp.*[n=2] |  |
